# ‘It's not magic’: A qualitative analysis of geriatric physicians' explanations of cardio‐pulmonary resuscitation in hospital admissions

**DOI:** 10.1111/hex.13212

**Published:** 2021-03-08

**Authors:** Anca‐Cristina Sterie, Laura Jones, Ralf J. Jox, Eve Rubli Truchard

**Affiliations:** ^1^ Palliative and Supportive Care Service Chair of Geriatric Palliative Care Lausanne University Hospital and University of Lausanne Lausanne Switzerland; ^2^ Service of Geriatrics and Geriatric Rehabilitation Chair of Geriatric Palliative Care Lausanne University Hospital and University of Lausanne Lausanne Switzerland; ^3^ Institute of Humanities in Medicine Lausanne University Hospital and University of Lausanne Lausanne Switzerland

**Keywords:** cardio‐pulmonary resuscitation, code status, CPR, explanations, geriatric patients, informed decision making, medical decision making, patient‐centred care, physician‐patient communication, shared decision making

## Abstract

**Background:**

Discussing patient preferences for cardio‐pulmonary resuscitation (CPR) is routine in hospital admission for older people. The way the conversation is conducted plays an important role for patient comprehension and the ethics of decision making.

**Objective:**

The objective was to examine how CPR is explained in geriatric rehabilitation hospital admission interviews, focussing on circumstances in which physicians explain CPR and the content of these explanations.

**Method:**

We recorded forty‐three physician‐patient admission interviews taking place in a hospital in French‐speaking Switzerland, during which CPR was discussed. Data were analysed in French with thematic and conversation analysis, and the extracts used for publication were translated into English.

**Results:**

Mean patient age was 83.7 years; 53.5% were admitted for rehabilitation after surgery or traumatism. CPR was explained in 53.8% of the conversations. Most explanations were brief and concerned the technical procedures, mentioning only rarely potential outcome. With one exception, medical indication and prognosis of CPR did not feature in these explanations. Explanations occurred either before the patient's answer (as part of the question about CPR preferences) or after the patient's answer, generated by patients' indecision, misunderstanding and by the need to clarify answers.

**Discussion and conclusions:**

The scarcity and simplicity of CPR explanations highlight a reluctance to have in‐depth discussions and reflect the assumption that CPR does not need explaining. Providing patients with accurate information about the outcomes and risks of CPR is incremental for reaching informed decisions and patient‐centred care.

**Patient contribution:**

Patients were involved in the data collection stage of the study.

## 1 INTRODUCTION

Physicians play a crucial role in informing patients about treatment options and in obtaining informed consent for treatment decision making; this is all the more important when it comes to life‐prolonging interventions such as cardio‐pulmonary resuscitation (CPR), which is regularly discussed in anticipation on hospital admission. While CPR for older hospitalized patients generally results are rather poor,^1^ many patients hold erroneous beliefs overestimating the survival chances and underestimating the risk of adverse outcomes, beliefs that underpin their wishes to undergo this procedure.^2^, ^3^ Choices in favour of CPR may also reflect the patients' impression that forgoing CPR is equivalent to choosing death over life.^4^

In order to express their wishes regarding CPR, patients must be aware of the circumstances in which CPR may be necessary, be aware of all options of treatment or action and be able to make sense of the potential outcomes of these options. Prior studies have shown that patient preference changes, when physicians provide adequate information about outcomes and discuss the patient's personal prognosis after CPR (eg, the risk of being in a dependent health state).^4^ Yet physicians report low confidence and high discomfort with the topic, especially due to possible ramifications towards discussing end of life.^5^ Physicians expect these discussions to be emotionally difficult for patients and thus avoid having them, especially when CPR is not medically indicated and they would have to explain this.^6^ Other barriers documented in discussing CPR include a lack of clinical knowledge, for example having inadequate understanding of CPR outcomes and experiencing difficulty in predicting patient trajectory and prognosis.^7^ Physicians themselves highlight a lack of training, peer guidance and role models or mentoring opportunities which would aid junior physicians in refining skills and would promote shared decision‐making approaches.^7^, ^8^

The establishment of an international gold standard on how CPR discussions should be conducted is difficult, given the very different cultural contexts and ethical legislation. Specific guidance exists for some countries, established by national authorities such as in the UK ^9^ and regional ones such as in Western Australia.^10^ Practice is also informed by guidance on ethical decision making.^11^ Professional development at a hospital level is another source of guidance on this topic. Overall, recommendations stipulate that health professionals should start by describing cardiac arrest and its causes, followed by a procedural explanation of CPR, supplemented by information regarding what happens if CPR is successful in preventing death. Health professionals should provide information about the risks of the procedure (cognitive sequelae, broken ribs) and present the chances of survival in terms of the patient's individual prognosis.^12^, ^13^, ^14^

Nevertheless, prior studies highlighted that CPR conversations with hospitalized patients still rarely follow these guidelines. Physicians primarily focus on CPR procedures, rarely referring to likelihood of cardiac arrest or outcomes after the procedure.^15^, ^16^, ^17^, ^18^, ^19^, ^20^ Instead of discussing personalized risk of cardiac arrest, the topic of CPR is introduced using disclaimers to normalize the need to discuss CPR and present it as part of the hospital routine.^15^, ^19^, ^20^ Furthermore, authors note the use of ambiguous language and euphemisms.^18^ CPR is frequently presented and framed as a default protocol, and little information is provided on options such as do‐not‐attempt‐to‐resuscitate (DNAR) or comfort measures.^15^, ^21^ Physicians refrain from questioning patients' understanding of the procedure, accept decisions as unilateral ^22^ and provide few opportunities for the patients to express their fears or concerns concerning end of life or CPR.^17^

Given the pivotal role that communication plays in making these medical decisions, it is important for researchers to explore the explanations given by physicians during actual CPR discussions and how patients develop an understanding and a preference in situations such as routine admission interviews. While revealing the scarcity of explanations employed by physicians, previous studies have failed to go beyond the content of these explanations and investigate when and how information is delivered.

Furthermore, to date, studies on CPR conversations at hospital admission mainly focussed on the general patient population ^16^, ^18^, ^21^ and on seriously ill^15^ or palliative care patients.^19^ There is little to no data‐driven information on how such conversations take place with geriatric patients who are admitted for rehabilitation, even though such discussions are also routine in this setting and the decision is less straightforward given the high heterogeneity of the geriatric population.

### 1.1 Aim

The aim of this research was to explore the circumstances in which physicians explain CPR as well as their content and the way these explanations are delivered to patients. In addition, we aimed to examine what makes such explanations relevant and how patients respond to them.

## 2 METHODS

### 2.1 Participants

The study population concerned patients transferred from geriatrics, internal medicine and traumatology/orthopedics services to the geriatric rehabilitation facility of the same Swiss university hospital. Informed consent to audio‐record their admission interviews was obtained 24‐48 hours prior to their transfer to the rehabilitation facility. Patients had no delirium or cognitive problems that would impact their decision‐making capacity, as determined by the physician's assessment of their medical file (Author 3 or 4). Patients who had been admitted to the same facility during the three months preceding their current admission were excluded. Companions were not included in the study as it is very rare for them to be present during the admission interview. Resident physicians who routinely conduct admission interviews were asked for their permission to audio‐record these interviews.

### 2.2 Data collection

Forty‐three physician‐patient admission interviews were audio‐recorded between June 2017 and January 2018. Both physicians and patients consented to the audio‐recording of their interviews and gathering of patient data from the medical files. Physicians and patients were told that the study aimed to investigate physician‐patient communication, without specifying the focus on CPR beforehand, so that bias was avoided and the interaction was as naturalistic as possible. Personal data were erased or anonymized, and patients' and physicians' voiceprints in the recordings were anonymized (electronically altered) using voice conversion software (Audacity) in order to maintain confidentiality. The audio recordings were preceded by ten ethnographic observations (Author 1).

Fifty‐one patients and 17 physicians gave oral and written consent to their admission interviews being recorded. Forty‐three conversations involved a discussion of the patient's resuscitation wishes. The parts of interaction which focused on CPR were selected and transcribed verbatim according to the Jeffersonian system ^23^ and checked for accuracy (Authors 1 & 2). The non‐transcribed parts of the interviews were listened to by the researchers to understand the context of CPR conversations.

The project was approved by the regional research ethics commission of the canton of Vaud (Switzerland) (2017‐00229).

### 2.3 Analysis

Transcripts were imported into the qualitative analysis software MaxQDA and read multiple times by Author 1, a sociologist experienced in conversation analysis, and Author 2, a researching psychologist experienced in thematic and discourse analysis, to identify an initial coding framework which focused on the components of the conversations. These coding frameworks were then compared, and the researchers checked that all conversation components were captured. All transcripts were coded into the major themes by Author 2. Themes within these categories were then developed inductively.

The transcripts were re‐read, and the broad‐level coding was checked within these major themes, before more specific themes were identified and transcripts were re‐read and coded. Authors 1 and 2 discussed these themes at each stage. The excerpts selected were also analysed through conversation analysis^23^ by Author 1 to identify the situations in which explanation sequences are occasioned and how patients orient to physician's explicative turns.

While the analysis was done in the original language (French), extracts were translated from French to English to allow comprehension of non‐French readers. Translations were done by native English‐speaking author 2 and validated using retranslation by native French‐speaking author 1. This is a literal translation which reflects the original language syntax structures. Italics are used in the transcript to differentiate the physician's explanations from the rest of the talk in order to improve readability. In the excerpts presented here, Cxx identifies the number of the conversation in our collection; Phy#X identifies the physician recorded; and Pat#X identifies the patient.

## 3 RESULTS

### 3.1 Context

Admission interviews involved taking a medical and social history, performing a geriatric assessment, and conducting a physical examination. Decisions about CPR code status were made during the interview; according to the ethnographic observations, this could also be revised by the medical team after the interview and/or rediscussed with the patient during the course of the hospitalization.

CPR was discussed in 43 of the 51 recorded admission interviews. Mean length of resuscitation conversations was 1 minute 45 seconds (SD = 96 seconds). Among the patients studied, 11 expressed the wish to be resuscitated, 20 did not want to be resuscitated, and 12 expressed uncertainty or were unclear about their wishes.

Patient information is described in Table 1.

**TABLE 1:** Patient characteristics

Patient demographic	Number (%)
Age	83.65
Reason for hospitalization	
Rehabilitation after surgery or traumatism	23 (53.5)
Deconditioning after urinary infection	3 (6.9)
Rehabilitation after septic choc	3 (6.9)
Geriatric rehabilitation (general)	2 (4.7)
Other	12 (30)
Service from which they are transferred	
Geriatrics	5 (11.6)
Internal medicine	21 (48.8)
Traumatology and Orthopedics	17 (39.6)
Existence of a prior code status	
Yes	11 (25.6)
No	25 (58.1)
Not available	7 (16.3)
Existence of a code status after the discussion	
Yes	13 (30.2)
No	29 (65.1)
Not available	1 (2.3)
Existence of advance Directives	
Yes	3 (6.9)
No	15 (34.9)
Not available	25 (58.2)

#### 3.1.1 Explanations

CPR conversations generally consisted of four phases: introduction, request for patient's preference, clarification and/or confirmation of the patient's preference, and closing.^20^ Explanations of CPR were present in the second or third phase, and this part of the conversation was selected for further in‐depth analysis. Four codes were created:

- explanations appearing in the physician's initial question;
- explanations appearing as clarification‐confirmation of the patient's preference;
- explanations appearing after patient doesn't specify a preference;
- no explanations.

Overall results are resumed in Table 2.

**TABLE 2:** Main results

Result	Number (%)	Example
Conversations containing explanations of CPR	23 (54%)	
Sequencing (first feature of CPR explanation)		
In the physician's question	8 (23%)	‘Phy: Would you wish for us to do a resuscitation, to do a cardiac massage to try to restart the heart’ (Excerpt 1, C22)
After the patient stated a preference, as a clarification or confirmation	11 (48%)	‘Phy: If the heart stops, what do we do? Pat: Nothing Phy: So, no intensive care, no tubes, no resuscitation?’ (Excerpt 4, C3)
After patient doesn't specify a preference	4 (17%)	‘Phy: In the case that the heart stops… Pat: I will be buried not cremated Phy: Would you want that we start resuscitation? That we try to restart the heart?’ (Excerpt 5, C48)
Content		
Procedure	13 (57%)	‘Phy: would you wish that we redo… that we try to make it work again, give an electric shock, put a tube eventually to sustain the lungs?’ (Excerpt 2, C44)
Technical aspects + Outcomes	9 (39%)	‘Phy: resuscitation, it's (2.9) it's something that is not magic, it's not enough to re‐press on a button, we need to re‐press on the heart, it restarts and everything is fine, I mean, in any case, the chances of success with a resuscitation are in any case very low’ (Excerpt 3 (C35)
Only outcomes	1 (4%)	‘Phy: if your heart stops… Pat: you have to let it stop Phy: you don't want us to try CPR? Pat: I'm 85, I don't expect any miracles Phy: Right, do you know what CPR is? Pat: Yes, yes. No, no, we drop it. Phy: All right. Why don't you want CPR? Pat: Why should I? Phy: You know that there are risks associated with CPR?’ (C9)

In 23 of the 43 conversations (54%), the physician explained what CPR entails. In 13 of these conversations, the explanations only concerned the technical aspects of the CPR procedure (such as cardiac massage, intubation, defibrillation) and additional tangent activities (calling an ambulance). In nine additional conversations, the physician provided explanations about the procedure and about its outcomes – risks and survival rate. In one conversation, the explanation concerned only the outcomes of the procedure.

Explanations occurred either before the patient's answer (as a part of the physician's initial question about CPR preferences) or after the patient's answer (combined with a request to confirm or clarify their wish or in response to the patient not specifying their preference).

(i) Explanation as part of the physician's initial question

The first instance in which CPR was explained was as part of the physician's initial construction of the question about the patient's CPR preferences (8/23 conversations; 35%).

In seven of these instances, the explanation was brief and focused on the procedure:

Excerpt 1 (C22)

Phy#3: (…) would you wish for us to do a resuscitation, to do a *cardiac massage to try to restart the heart?*

Explanations which occurred as part of the question came about before the patient had an opportunity to provide input and were therefore not prompted or invited by the patient.

In two cases, CPR was referred to in the question only by mentioning the elements it involves, but not explicitly.

Excerpt 2 (C44)

Phy#15: If something happens to your heart, if it stops working all of the sudden or the lungs stop functioning all of the sudden, would you wish *that we redo… that we try to make it work again, give an electric shock, put a tube eventually to sustain the lungs?*

Avoiding medical terminology such as ‘resuscitation’ may render the information more easily understandable and more informative for the general population. Referring to an 'electric shock', while it could be experienced as disturbing, is closer to a phenomenological experience of day‐to‐day life than a reference to 'defibrillation', as is 'a tube to sustain the lung' compared to 'intubation'.

Six of the eight explanations occurring while asking the CPR question were exclusively procedure‐oriented and provided no information on chances of survival or potential complications. Only two explanations provided more detail.

Excerpt 3 (C35)

Phy#14: (…) What would you wish to do if your heart stopped? (1.4) Uhmmm (1.1) In the sense that, resuscitation, it’s (2.9) *it's something that is not magic, it’s not enough to re‐press on a button, we need to re‐press on the heart, it restarts and everything is fine, I mean, in any case, the chances of success with a resuscitation are in any case very low* (…)

In this instance, the physician asks a direct question about the patient's wishes in case of cardiac arrest, which is information‐void as it does not provide any material which would aid the patient in making the decision. Only after the patient provides no response to this question ('(1.4) Uhmmm (1.1)'), the physician goes on to reengage with the topic by firstly emphasising that CPR is not straightforward ('magic' 'it's not enough to…'; 'we need to…'). The procedure‐focussed details about the actual steps of CPR delivery and its consequences (‘we need to re‐press on the heart, it restarts and everything is fine’) are an ironic, throw‐away explanation. This mitigates the commonly held belief that CPR might be as simple as 'pushing a button', as the physician later concretely states that chances of this happening are ‘very weak’. This presents CPR as a complicated intervention with little chance of success. This ‘shaded’ framing^24^ discretely presents CPR as something undesirable. These explanations are therefore not only information‐laden but may convey, through word choice (reference to 'magic') the physician's opinion regarding CPR not being medically indicated. Nevertheless, the basis for this normative evaluation is not shared.

(ii) Explanation after the patient's expresses their preference

In 11/23 instances (48%), additional information about CPR was given in the form of a request after the patients have already indicated their preferences, pursuing a clarification or confirmation of this choice. These explanations were again generally brief and focused on procedures.

In excerpt 4, the physician's open question generates a straightforward answer that still needs to be unpacked and translated in terms of wanting or not wanting CPR:

Excerpt 4 (C3)

Phy#7: (…) in our entry file we have standard questions that probably someone has already asked at the main hospital. (…) If the heart stops, what do we do? (…)

Pat: Nothing.

Phy#7*:* *So, no intensive care, no tubes, no resuscitation?*

Pat: Listen, resuscitation, it depends what you mean by resuscitation?

Phy#7*:* *Resuscitation, it could be a simple cardiac massage, but it could also be the ambulance coming, we put lots of tubes into you, we start to…*

*Pat:* *Yes, but the cures, sometimes it's also the stroke afterwards…*

Phy#7*:* *That’s true, we don’t always know what will happen after. No one can guarantee you that*.

The patient responds to the question with a direct ‘nothing’, providing no elaboration or justification for the response. The physician then clarifies what doing ‘nothing’ means, that is, the absence of CPR procedures. The patient responds to this clarification request with a question himself asking the physician to clarify the meaning of resuscitation, indicating that the previous explanation was insufficient. The physician then provides a range of possible interventions from ‘simple cardiac massage’ to ‘lots of tubes’. The patient initiates a discussion about the possible complications after resuscitation, which invites the physician to highlight the uncertainty of the outcomes. In this excerpt, the physician acknowledges the uncertainty surrounding a hypothetical resuscitation; however, despite the patient's initiation of this discussion, he does not take up the opportunity to explain the consequences and what they would mean for that patient and their life.

Throughout the data, open questions generated generic (such as in excerpt 4) or metaphorical responses (such as ‘I’m a fighter’ or ‘As long as there's life, there's hope’), which is unsurprising as these questions do not refer explicitly to CPR. The physicians subsequently translate the patient's answer into a decision about CPR, whereby clarification sequences are useful in more nuanced responses. While this format provides the patient with complete autonomy to select a course of action, it requires the patient to be knowledgeable about the available options.^20^

In two conversations of this sub‐corpus, the clarification was offered by the physician after the patient provided a clear answer. In one of the conversations, the physician pursued a clarification of the reasons for which the patient chose to forego CPR (as a verification of his understanding of the procedure). In the other conversation, the clarification provided information on side‐effects and occurred after the patient expressed a preference in favour of CPR.

(iii) Explanation after the patient doesn't specify a preference

In 4/23 instances (17%), the explanation was provided as an uptake of a response that did not contain a wish, either because the patient appeared not to understand the question or was unsure about it.

In the following conversation, as the physician starts to enquire about CPR, the patient talks about funeral arrangements; as the topic becomes clearer, the patient admits not having thought about it. This repetitive lack of expressing a preference makes the physician provide more explanations about CPR.

Excerpt 5 (C48)

Phy#13: It happens rarely, but in the case that the heart stops…

Pat: I've done (…) I am to be buried in (name of cemetery), I will be buried not cremated (…) it's payed, yes.

Phy#13: All right. But if you are here with us and the heart stops.

Pat: Yes.

Phy#13: Would you want that we start resuscitation? *That we try to restart the heart*?

Pat: Oh this? (…) I've never done this, no.

Phy#13: But have you already thought about the question?

Pat: Not even

v#13: Do you know what it is, resuscitation?

Pat: Yes, yes.

Phy#13: *We start to massage, if the heart doesn’t restart, we must put tubes in the throat for artificial breathing*. Would you like us to do that?

Faced with the patient's initial deviation from medical procedures to be undertaken in case of heart arrest to funeral arrangements, the physician reengages in the topic, making the topic of CPR more explicit and providing a brief explanation.

The patient avows that she has never documented her wishes regarding CPR before nor thought about the topic. This prompts the physician to question her comprehension regarding CPR, which results in a more detailed explanation.

Despite the patient's repetitive assertion (‘yes yes’), the physician continues with an explanation, possibly due to the unclear nature of the patient's previous response. Compared with the minimalistic description provided initially ('heart stops', 'restart the heart'), this comparatively elaborated explanation offers more details ('massage', 'put tubes'), yet again remains procedural, with no discussion about potential complications or chances of success.

Excerpt 6 displays another example of a patient not providing their wish; this time, the patient tries to delegate the decision to her son. When the physician insists on the patient making the decision, she admits not knowing what to answer, prompting further explanation:

Excerpt 6 (C42)

Phy#15: (…) if the heart all of the sudden, it stops, the lung,

Pat: Yes, it's my son [who decides].

Phy#15: Yes, but I mean, yourself, you wish us to do a resuscitation? That we try to restart the heart?

Pat: I don't know, I don't know about this. I don't know at all.

Phy#15: *So, let's say that when we have a cardiac attack which… which… in which the heart stops. It's quite serious as complication, so what we have to do is use perhaps some electric current to shock the heart, to put a tube to support the lung. Sometimes one recovers well, other times we can have side effects, or not recover or have side effects. This is something that is unpredictable*.

Pat: Yes, we can't know.

Phy#15: *We can't say what a person… we know that the longer we try to resuscitate…*

Pat: Yes, yes.

Phy#15: *The more possible it is that complications arise, but again we can't know…*

Pat: No, we can't know, of course.

Phy#15: So, if it happened and if there was an attack, do we try to resuscitate?

Pat: Yes try, yes try.

The physician's explanation is an elaboration of her initial question and covers several aspects. Firstly, the circumstances in which resuscitation becomes relevant ('cardiac attack', 'the heart stops', 'serious complication'); followed by an explanation of the procedure in relative detail ('use electric current', 'shock the heart', 'put a tube'); and finally the outcomes (recover well', 'complications', 'not recover', 'unpredictable').

While the patient acknowledges throughout the physician's explanation (‘yes we can't know’; ‘yes, yes’, ‘no, we can't know, of course’), she resists giving a definitive answer and expressing her preference. The physician persists highlighting the uncertainty of the outcomes, requesting a more definite response from the patient. After three attempts, the physician finally reformulates her initial question regarding CPR, which the patient accepts.

#### 3.1.2 No explanation of CPR

In 20/43 conversations (48%), ‘resuscitation’ does not get unpacked at any point in the conversation and no explanations are provided of what CPR means. In two of these cases, the question was asked in a manner in which the response is implied and the patient is asked to confirm this response,^20^ and in another 13 cases, the patient provides a straightforward preference which leaves little to be clarified; the decision is therefore documented as it is.

Excerpt 7 (C2)

Phy#6: And just one question that we ask each patient who is hospitalized here, if your heart it stops, do you wish that we resuscitate you or not at all?

Pat: Oh yes of course.

Phy#6: All right.

This immediate and definite response may function to end the possibility for further discussion, and therefore explanation, on the subject of CPR.

In 3 instances, this assertive decision was accompanied by patients indicating that they had already answered these questions elsewhere, thus acting as a deterrent for further explanation.

Excerpt 8 (C6)

Phy#3: (...) if something very very serious happened to you, what would you wish that physicians do or not do?

Pat: I’ve already responded to this question multiple times.

Phy#3: At the hospital.

Pat: Yes.

Phy#3: Yes.

Pat: No, no aggressive treatment. I wouldn't want that.

Phy#3: All right, okay.

Pat: If there's nothing else to do, if I become a vegetable, then no.

Phy#3: All right. Okay. It's always useful for us to know in what state of mind you are.

Compared to responses to open what‐questions (excerpt 4), this patient asserts her position as a knowledgeable and active participant ('I've already responded to this question'), backing up her preference with reference to unreasonable life‐prolonging therapies ('aggressive treatment'). She also explains as reason for her choice not wanting to ‘become a vegetable’. The fact that CPR was discussed previously, combined with the patient's assertiveness and explanation, reduces opportunities for an extended explanation of CPR.

## 4 DISCUSSION

Consistent with previous research,^15^, ^18^, ^21^ explanations of CPR in our data set are scarce, and when existent, they are generally simplistic and procedure‐focussed. Our findings indicate that explanations occur when they appear as part of the question whether the patient wants to be resuscitated or not, and when confronted with patient wishes which needed clarification. These clarifications shed light on the physician's role in translating patient's responses into medical decisions about CPR. In the rare cases where the risks and outcomes of CPR were discussed, they were occasioned by the patient, for example through their misunderstanding of the question or uncertainty. These explanations were lengthier, integrating aspects concerning the occurrence of cardiac arrest, the CPR procedure and its outcomes. Except for one case, explanations did not refer to medical indication or chances of survival, an information which is particularly relevant in the case of geriatric populations.

References about the expectation that CPR has been discussed present the topic as already known to the patient and work to display it as less threatening. Prior research has already shown that this interactional device also assists recipients in making a medical decision.^25^ However, assuming this precedence may also prevent patients who have not done so or don't remember having done so, from stating otherwise. This is problematic for obtaining an informed decision as patients need a fuller understanding of what they are consenting to. This phenomena also begs the question of whether making an informed decision really is the goal of these conversations or whether there is an underlying assumption that a decision has already been made and the task is simply to retrieve this decision and document it. Even if this is the case, in a geriatric population circumstances may have changed, preferences should be continuously rediscussed and reasons explored. We would encourage physicians to begin by investigating whether patients have discussed CPR before and *what* they have been told about it so that they can structure explanations according to the needs of each patient.

The lack of explanations in almost half of conversations and their brevity reflect the taken‐for‐granted assumption that CPR is understood by everyone and therefore does not warrant explanation and unpacking. The choice in vocabulary is also of interest. The use of lay words (‘electric shock’ vs ‘resuscitation’) or catch phrases (‘it's not magic’) instead of medical terminology has the benefit of bringing the subject closer to the patient's experience but it may also open the way to wrong associations. Alongside information retrieved from media sources such as TV drama, this influences the patient's wish to undergo the procedure.^26^, ^27^

It is, however, important to note that the setting in which this research was conducted may have restricted opportunities for in‐depth discussions: not only is time limited, but physicians and patients just meet each other for the first time, rendering such conversations difficult. Furthermore, as patients were all transferred from another unit in the main hospital, there is reason to believe that CPR had been discussed before and a new discussion within a short time frame might be obsolete. As such, we also encourage hospital physicians to look unto CPR conversations as an iterative process, in which the patient is offered several chances for feedback and for reviewing their preferences over time.

Several studies highlight the inadequacy of information provided by physicians in CPR discussions with hospitalized patients, across cultures.^15^, ^17^, ^18^, ^21^ Physicians need to assess which information is pertinent for each patient and to tailor this to meet the objectives of the interaction. The flow of the interaction is thus capital: physicians must be receptive to patient cues and opportunities to discuss CPR in relation to the patient's specific condition and as more than an abstract concept that ‘probably won't happen’. Our data highlight many missed opportunities to discuss understandings of ‘taken‐for‐granted’ concepts, to develop a shared understanding of what CPR means, its potential outcomes, and to enter into a discussion about what these outcomes would mean for the patient's life and what the patient would consider acceptable or unacceptable conditions in which to live. The lack of more in‐depth information exchange may lead to decisions that, even when CPR is medically viable, are not in the best interest of the patient. In‐hospital CPR conversations could thus be a good starting point for a longitudinal advance care planning process that may be continued after discharge.^28^

Our data also show that certain situations make explanation‐giving more difficult, for example when patients seem very convinced about their wish or assume they are particularly well informed about CPR. Given that a lot of false information about CPR is propagated,^2^ we argue that even in cases in which patients seem knowledgeable, there is an ethical requirement for the physician to learn more about the patient's understanding of CPR as the basis for his or her wish.

Finally, we want to point out that alternative options to CPR are never described or explained by physicians but referred to at most as 'not doing CPR' or 'letting the heart stop'. We argue that more disclosure about not engaging in CPR, in the form of more open discussions about death and dying, is also as much a part of informed decision making as information about CPR itself.

### 4.1 Limitations

The recordings come from one rehabilitation hospital whereby the patients have been transferred. This homogeneity of interactions has produced a narrow account of interactions discussing resuscitation wishes. These results are specific to the Swiss context, and therefore, while general recommendations about supporting patients in making informed decision can be applied to contexts which are culturally similar, further detailed research is necessary in other countries.

## 5 CONCLUSION

With a high risk of adverse outcomes, CPR is of little value to geriatric patients suffering from multiple morbidities.^29^ Ethical and legal principles stipulate that physicians are not required to offer futile interventions that may generate further suffering ^30^; this is particularly pertinent for life‐sustaining and prolonging therapies such as CPR. For this reason, some authors insist that CPR should not be discussed with patients who would not benefit from it.^9^ Here, we argue that these discussions are useful even with patients for whom CPR would not be initiated. Firstly, due to the misconception within the general patient population that CPR is standard procedure regardless of condition, and it would be unethical to let them believe so. Secondly, a discussion about CPR would help patients become more knowledgeable about their medical trajectory and gain information that would allow them to understand why CPR is not medically beneficial for them, while being reassured that everything will be done to maintain their quality of life and death. Thirdly, discussions about CPR frequently elicit patients' concerns about their life and end of life; knowing more about patient goals and values is precious for establishing better physician‐patient relationships and is incremental for offering patient‐centred care, as it allows for shared understanding, and therefore a more even distribution of power and responsibility, from the patient and the physician.^31^ Nevertheless, these discussions must go further than *questions* about preferences and must discuss these preferences in light of medical indication. In light of these factors, CPR should be broached in a more suitable environment, before hospitalization occurs, such as during advance care planning, which offers more time to approach life values and goals, and relay information.^32^, ^33^ In this way, CPR preference can be revisited at each hospital admission, building on the information provided previously.

The conduct of CPR discussions influences patient understandings of the purpose of the interaction and has ethical implications for decision making. Navigating these conversations necessitates adequate medical knowledge and communication skills. The scarceness of CPR explanations seen here highlights physician reluctance and is reflective of the sensitive nature of the subject as well as taken for granted assumptions that CPR is something that is understood by everyone. This is problematic in medical interactions as it does not provide space for patients to ask questions and also limits opportunities for physicians to provide explanations and enter into more in‐depth discussions with patients. Explaining the outcomes and risks of CPR is an essential step in supporting patients to make informed and autonomous decisions, which is fundamental to achieving patient‐centred care and one of the cornerstone principles of medical ethics. Creating environments in which patients feel that they have the agency to ask questions and participate actively, and in which comprehensions of medical procedures such as CPR are co‐constructed and understood are essential. De‐construction of commonly held beliefs about CPR and explanation of medical terminology are thus indispensable. Appropriate training is vital for this task.

## CONFLICT OF INTEREST

We have no conflict of interest to declare.

## Data Availability

The data that support the findings of this study are available on request from the corresponding author. The data are not publicly available due to privacy or ethical restrictions.
